# Determination of Curcuminoids in Turmeric Dietary Supplements by HPLC-DAD: Multi-laboratory Study Through the NIH-ODS/NIST Quality Assurance Program

**DOI:** 10.1093/jaoacint/qsaa069

**Published:** 2020-11-01

**Authors:** Elizabeth M. Mudge, Paula N. Brown, Catherine A. Rimmer, Melissa M. Phillips

**Affiliations:** 1 British Columbia Institute of Technology, Natural Health & Food Products Research, Burnaby, BC, Canada; 2 National Institute of Standards and Technology, Material Measurement Laboratory, Chemical Sciences Division, Gaithersburg, MD, USA

## Abstract

**Background:**

Turmeric is a medicinal herb containing curcuminoids, used as quality markers in dietary supplements. In 2016, an AOAC First Action *Official Method*^SM^ was adopted for quantitation of curcuminoids and requires multi-laboratory reproducibility data for Final Action status.

**Objective:**

To collect reproducibility data for the quantitation of curcuminoids in dietary supplements through the National Institutes of Health Office of Dietary Supplements/National Institute of Standards and Technology Quality Assurance Program (QAP).

**Method:**

Laboratories that participated in the QAP by following the *Official Methods of Analysis*^SM^ Method 2016.16, submitted data for ten turmeric products. The data were analyzed for mean, repeatability, and reproducibility standard deviations, repeatability, and reproducibility.

**Results:**

The initial data collection resulted in insufficient replicates (five) for each test sample to determine reproducibility, therefore laboratories were provided additional materials resulting in an incremental data approach. For homogenous products, reproducibility for curcumin ranged from 3.4 to 10.3%, bisdemethoxycurcumin with reproducibility ranging from 6.4 to 14.8%, and demethoxycurcumin ranging from 5.6 to 9.9%. The method was unsuitable for the quantitation of curcuminoids in complex smoothie products, products containing microbeads, or tinctures based on interlaboratory variances. Recommendations were provided for future multi-laboratory studies performed through QAPs and incremental approaches.

**Conclusions:**

Method 2016.16 is suitable for the quantitation of curcuminoids and should be adopted for Final Action status for single and multi-ingredient dietary supplements containing dried roots, dried powders/extracts in bulk material, capsules, and softgels.

**Highlights:**

Reproducibility for Method 2016.16 was collected through a non-traditional incremental data multi-laboratory study. The method is suitable for quantitation of curcuminoids in most common dietary supplements.

Turmeric, *Curcuma longa* L., is a perennial herb originating from India and is in the family Zingiberaceae. The rhizomes of turmeric are thick and bright yellow due to the presence of curcuminoids, which are associated with the anti-inflammatory and antioxidant properties of the roots (1–3). Turmeric has been used in Aryuvedic and traditional Chinese medicine and is a commonly traded spice. With an increased demand for traditional medicines, many herbal ingredients are traded in bulk as highly-processed dried powders, lacking morphological characteristics of the original root material (4). In the absence of the unprocessed root material to confirm identity, incidences of adulteration of turmeric medicinal products with commercial dyes to enhance the yellow color is known to occur (5–7). In order to detect adulteration of products and to ensure product quality and safety, fit-for-purpose analytical methods are required to monitor active ingredients in herbal ingredients and finished products.

In 2015, as an activity of the Stakeholder Panel on Dietary Supplements, AOAC INTERNATIONAL established a working group to develop standard method performance requirements (SMPRs) for curcuminoids in turmeric. The working group published SMPR 2016.003 for the quantitation of three curcuminoids; curcumin, demethoxycurcumin, and bisdemethoxycurcumin, in dietary ingredients and finished products alone or in combination with other dietary ingredients (8). A call for methods led to the acceptance of First Action *Official Methods of Analysis*^SM^ (OMA) 2016.16 (9). The original method was optimized and validated using statistically guided protocols for all three curcuminoids in turmeric roots, extracts, and commercial products (10, 11).

This work summarizes the collection of reproducibility data, which is mandatory for adoption to a Final Action OMA. A collaboration between the Natural Health & Food Products Research Group (NRG) at the British Columbia Institute of Technology (BCIT) and the Chemical Sciences Division (CSD) at the National Institute of Standards and Technology (NIST) was initiated to collect reproducibility data in an incremental approach through NIST’s Dietary Supplement Laboratory Quality Assurance Program (DSQAP), funded by the National Institutes of Health (NIH) Office of Dietary Supplements (ODS). Participating laboratories in the program could voluntarily analyze samples using the OMA or use an in-house method. Data collected from laboratories that reported using the OMA were used to determine reproducibility for curcuminoid analysis by HPLC diode-array detection.

## METHOD

### Multi-Laboratory Study Design and Participating Laboratories

The NRG at BCIT and the CSD at NIST jointly organized the multi-laboratory study. Laboratories were recruited through their past participation in the NIH-ODS/NIST DSQAP. The participants could choose to analyze samples according to OMA 2016.16, or another method of their choice. Participating laboratories were provided with the official method protocol and test samples. Only data collected by laboratories using OMA 2016.16 was used to determine method reproducibility. The original study design was to provide participating laboratories with a sub-set of samples to evaluate method performance with a minimum of eight replicate measurements (laboratory results) per test material. Due to the split-sample design and the fact that not all laboratories reported using OMA 2016.16 for determination of curcuminoids, a second round was conducted in which laboratories were sent additional untested samples from the study. Therefore, this multi-laboratory study was evaluated using an incremental approach over time. The results were evaluated using AOAC guidelines for collaborative studies (12).

### Sample Details

Two NIST SRMs were provided, SRM 3299 Ground Turmeric (*Curcuma longa* L.) Rhizome and SRM 3300 Curcumin Extract of Turmeric (*Curucma longa* L.) Rhizome, in addition to eight commercial products with varying matrices and ingredients. Capsules and softgels were prepared by combining the contents of several bottles of the same lot number for each test sample. Subsamples of 20 capsules or softgels were placed into sealed aluminum packets and provided to the laboratories. Liquid and dry powders were combined, homogenized, and sub-sampled into individual vials or aluminum packets. The details of the test materials are summarized in Table 1. Samples were provided to laboratories with labels from A to J. Samples A through H were provided in triplicate and laboratories were instructed to prepare one sample and report one value per packet or vial provided. Samples I and J were provided as a single packet and laboratories were instructed to prepare three samples and report three values from the single packet provided.

### Analysis and Data Reporting

Laboratories that participated in the DSQAP and followed OMA 2016.16 submitted their data to NIST through their online submission form. The data was summarized and verified to ensure accuracy of the reported data.

### Validation Data Analysis

Outliers were identified and removed based on results of Cochran’s C tests and single and double Grubbs’ tests. The calculation for mean, repeatability, and reproducibility standard deviations (SD_r/R_), repeatability (RSD_r_), and reproducibility (RSD_R_) were calculated based on AOAC guidelines using Microsoft Excel.

### HPLC-UV-Vis Method

The protocol for the method of analysis provided and used by all participating laboratories was as follows:

- *Principle*.—Samples are extracted with methanol using a wrist-action shaker. Dilution of highly concentrated samples may be required prior to LC analysis. Quantitation is performed with external calibration against reference standards for curcumin, demethoxycurcumin, and bisdemethoxycurcumin.
- *Apparatus*.—
  1. *Analytical balance*. —Sensitive to at least 0.0001 g.
  2. *Grinder*.—Retsch or equivalent centrifugal mill fitted with a 0.25 mm screen.
  3. *Volumetric pipet*.—Class A, 25 mL.
  4. *Centrifuge tubes*.—Conical polypropylene tubes, 50 mL.
  5. *Vortex mixer*.
  6. *Centrifuge*.—Benchtop with rotor for 50 mL conical tubes, maximum 21,000 × *g*_n_.
  7. *Shaker*.—Wrist action.
  8. *Micropipettors*.—Capable of pipetting 200 μL and 1000 *μ*L.
  9. *LC vials with caps*.
  10. *Volumetric flasks*.—Class A, 10 mL.
  11. *LC system.—*Agilent HP 1200 (Agilent Technologies, Santa Clara, CA, USA) with binary pump operating at 400 bar, temperature controlled column, autosampler, and UV-Vis diode array detector.
  12. *Chromatographic column*.*—*Kinetex C18, 2.6 *μ*m, 2.1 × 30 mm (Phenomenex, Torrance, CA, USA).
  13. *Syringes*.*—*Disposable luer-lok, 3 mL.
  14. *Syringe filters*.*—*Polytetrafluoroethylene (PTFE) membrane, 0.2 *μ*m.
  15. *Bottles*.*—*LC solvent bottles, 1 L.
- *Chemicals and reagents.—*
  1. *Curcumin (CUR) primary standard*. *—*Product No. ASB-00003926 (ChromaDex, Irvine, CA, USA).
  2. *Demethoxycurcumin (DMC) primary standard*. *—*Product No. ASB-00004230 (ChromaDex).
  3. *Bisdemethoxycurcumin (BDMC) primary standard*. *—*Product No. ASB-00004231 (ChromaDex).
  4. *Water*.*—*Nanopure deionized, or equivalent.
  5. *Methanol*.*—*HPLC grade.
  6. *Acetonitrile*.*—*HPLC grade.
  7. *Formic acid*.*—*HPLC grade.
- *Preparation of solutions and standards.—*
  1. *Curcumin stock solution, 1000 μg/mL (CUR-1000)*.—Weigh an amount of curcumin primary standard equivalent to 10.0 mg after correction for purity and place in a 10 mL volumetric flask. Fill to volume with methanol, mix, and store in a screw top vial at −20°C for up to 4 weeks.
  2. *Demethoxycurcumin stock solution, 1000 μg/mL (DMC*-*1000)*.*—*Weigh an amount of demethoxycurcumin primary standard equivalent to 10.0 mg after correction for purity and place in a 10 mL volumetric flask. Fill to volume with methanol, mix, and store in a screw top vial at −20°C for up to 4 weeks.
  3. *Bisdemethoxycurcumin stock solution, 1000 μg/mL (BDMC-1000)*.*—*Weigh an amount of bisdemethoxycurcumin primary standard equivalent to 10.0 mg after correction for purity and place in a 10 mL volumetric flask. Fill to volume with methanol, mix, and store in a screw top vial at −20°C for up to 4 weeks.
  4. *Mixed calibration standards.—*Prepare the mixed calibration standards daily. Calibration standards concentrations are described in Table 2 using the dilutions in methanol as described in OMA 2016.16.
  5. *LC mobile phase A*.*—*Dilute 1 mL formic acid with 999 mL water to make 0.1% formic acid in water.
  6. *LC mobile phase B*.*—*Dilute 1 mL formic acid with 999 mL acetonitrile to make 0.1% formic acid in acetonitrile.
- *Preparation of samples*.*—*
  1. *Homogenization of solid samples.—*Grind dried turmeric rhizome in a centrifugal mill with 0.25 mm screen. Combine 20 dosage units of bulk extract, capsules, or tablets and grind. Tablets can be ground using a coffee grinder. Combine the contents of 20 dosage units of softgel capsules and grind. Store ground samples in polypropylene tubes at room temperature protected from light. Mix well before removing test portions.
  2. *Extraction of solid test portions*.*—*Prepare all test portions in triplicate. For ground dried rhizomes, bulk extracts, capsules, or tablets, accurately weigh 75 mg into a 50 mL centrifuge tube. For ground softgel capsule contents, accurately weigh 200–300 mg into a 50 mL centrifuge tube. Add 25 mL methanol and vortex mix for 30 s. Shake on a wrist-action shaker for 15 min at room temperature. Centrifuge at 4500 *g*_n_ for 5 min. Filter 1 mL aliquot through a syringe filter into an LC vial. Store LC vial at 4 °C protected from light until analysis.
  3. *Preparation of tinctures*.*—*Prepare all test portions in triplicate. Mix tincture by inversion several times. Dilute 100 *μ*L tincture with 900 *μ*L methanol and vortex mix. Filter through a syringe filter into an LC vial. Store LC vial at 4 °C protected from light until analysis.
- *LC analysis*.*—*
  1. *Setup*.*—*Set detector to 425 nm, column temperature to 55 °C, injection volume to 0.8 μL, and flow rate to 1.4 mL/min. Program the gradient as follows: 0 to 1 min: 28% B; 1 to 2 min: 28–30%B; 2 to 4 min: 30% B; 4 to 4.1 min: 50% B; 4.1 to 6 min: 50% B (1.75 mL/min); 6.0 to 8.5 min 28% B. Equilibrate the column with the starting conditions of 28% mobile phase A.
  2. *Procedure*.*—*Make single injections of one set of calibration standards (stds 1–7). Make single injections of each LC sample. After approximately every 10 sample injections, and at the end of the run, re-inject one of the calibration standards for quality control purposes.
- *Calculations*.*—*
  1. *Calibration standards.*—Measure peak areas for CUR, DMC, and BDMC in the set of calibration standards and re-injected calibration standards. Ensure that the re-injected calibration standard peak areas are within 5% of the initial calibration standard peak areas.
  2. Construct a plot of analyte concentration (*x*-axis) versus individual peak areas (*y*-axis) for CUR, DMS, and BDMC. Use least squares analysis to determine the slope, intercept, and correlation coefficient (r^2^) of the best-fit line for each analyte
  3. *Unknown samples*.—from the standard curves and the peak areas of each analyte in the samples, calculate the concentration of CUR, DMC and BDMC in each sample solution. If the peak area of any analyte if above the standard curve for that analyte, dilute the extrat 1/10 or 1/20 in methanol, filter, and repear the analysis for that extract.

$$\;\text{curcuminoid}\;\text{concentration}\;\left(\frac{\text{mg}}{\text{L}}\right)\;=\frac{\;\text{curcuminoid}\;\text{peak}\;\text{area}\;-\;\text{intercept}\;\text{of}\;\text{linear}\;\text{regression}\;}{\;\text{slope}\;\text{of}\;\text{linear}\;\text{regression}\;}$$

- Calculate the amount of curcuminoid in the original sample as
  $$\text{curcuminoid}\;\text{in}\;\text{original}\;\text{sample}\;\left(\frac{\text{mg}}{\text{g}}\right)\;=\frac{\;\text{concentration}\;(\text{C})\;\times \;\text{volume}\;(\text{V})}{\mathrm{mass}(\text{W})}\times \text{D}$$
  where C = the concentration of analyte from the standard curve (mg/L), V = the extract volume (0.025 L), W = the weight of the test portion (g), and D = the dilution factor.
  Note: Certain commercial equipment, instruments, or materials are identified in this publication to adequately specify the experimental procedure. Such identification does not imply recommendation or endorsement by the authors or their respective institutions, nor does it imply that the materials or equipment identified are necessarily the best available for the purpose.

## Results and Discussion

A total of 34 laboratories volunteered to participate in the curcuminoid analysis through the DSQAP (13). To optimize sample distribution, each lab was provided with a subset of samples including the two NIST SRMs and four additional commercial matrix test samples for the multi-laboratory evaluation. Laboratories specified their method of analysis when submitting results and only laboratories that submitted results for all materials using OMA 2016.16 were included in the reproducibility evaluation. In total, nine laboratories met the requirements resulting in five replicates for each test sample, which was insufficient for determining laboratory reproducibility (12). All nine participating laboratories agreed to receive and test the additional matrices to finalize the data collection resulting in nine replicates for each sample. One exception occurred in one participating laboratory due to a mix up of samples G and I, resulting in eight replicates obtained for these samples. The initial round of samples was sent to laboratories in September 2017 and the second round in July 2018.

The reported curcuminoid contents determined by each laboratory are summarized in Tables 3–5 for CUR, BDMC, and DMC, respectively. Data found to be outliers based on Cochran and Grubbs’ tests are noted with stars and were removed from the reproducibility calculations. Plots of each laboratory result for each curcuminoid in each test sample are summarized in Supplemental Figures 1–10. Within-laboratory performance determined through standard deviations of each curcuminoid in the NIST SRMs and the majority of the commercial test samples indicated that all laboratories had acceptable repeatability when implementing the methodology. Unlike other types of collaborative studies, this incremental approach through the QAP does not provide practice materials to ensure the performance of the laboratory prior to inclusion in the study. The NIST SRMs were used as an alternative to verify laboratory performance. The SRM values compared to the average obtained from the multi-laboratory validation are within expected ranges as described in Table 6.

The overall reproducibility data obtained through this study are summarized in Table 7. Reproducibility appears to be higher for BDMC compared to the other analytes and is slightly higher than the ranges provided in the SMPR in most matrices. It should be noted that BDMC is the least concentrated curcuminoid in all samples and in several products is close to the lower limit of the analytical range as specified in the SMPR. The concentration of BDMC in sample D is below the required concentration range specified in the SMPR. For the samples within the SMPR concentration range, the reproducibility for BDMC ranged from 6.4 to 17.2%. Many products evaluated in this study are multi-ingredient commercial products with the potential to be heterogeneous. Reproducibility was best for homogenous samples such as single-ingredient products and SRMs, while multi-ingredient and heterogeneous products had higher RSDs. For this study, participants were not provided with calibration standards, as would occur in most traditional collaborative studies. Variability in the purity of calibration standards used by each laboratory may increase the between-laboratory variance being captured by the reproducibility, thereby increasing the range of observed reproducibility values which was not anticipated when the SMPR was established.

SMPR 2016.003 for curcuminoid quantitation provided a list of potential interfering dietary ingredients which must be considered to determine the method applicability (8). While several of these were evaluated during the original single-laboratory validation, additional ingredients were included in the multi-laboratory study for further verification. Products used in this multi-laboratory study included black pepper (*Piper nigrum*), ginger (*Zingiber officinale*), lutein, zeaxanthin, and magnesium, which were specified in the SMPR as potential interferences. Also evaluated were products including resveratrol, alpha-lipoic acid, coconut oil, lemon balm, and gota kola leaf. A full list of the additional ingredients has been summarized in Table 1. Based on the feedback provided by participants, no chromatographic interferences were observed from these ingredients.

A comparison of the reproducibility of samples E and F indicate limited impact on quantitation of curcuminoids from the addition of *P. nigrum* (black pepper) extract. The reproducibility of curcumin determination in these two products was 3.4 and 7.7%, with lower variance at higher concentrations even in the presence of black pepper extract. These data indicate that OMA 2016.16 performs similarly on multi-ingredient products and single-ingredient products. Microbeads present in sample G were visually apparent, and increased variability was observed in the data between laboratories for this sample. Therefore, this method as validated is not suitable for products with microbeads as additional homogenization would be required during the sample preparation.

As expected, the best comparability between laboratories was observed for the NIST SRMs, which have undergone rigorous homogenization steps. The commercial products utilized in this study did not undergo the same homogenization. Capsules and softgels were pooled from a single lot from several bottles, but the contents were not homogenized prior to distribution. Participating laboratories were instructed to thoroughly mix the contents of each packet of capsules or softgels as described in OMA 2016.16 prior to removal of a test portion for analysis, but heterogeneity between replicate samples of the commercial products may still have contributed to increased variability. Given the observed reproducibility of < 10% for the determination of CUR and DMC in the NIST products, acceptable performance of this method can be confirmed for homogeneous products.

The reproducibility for the tincture was not within expected ranges. Some laboratories may have incorrectly calculated dilutions, as the data appeared to range by factors of 2 to 10. A limitation of the incremental approach through the QAP was the inability to check the raw data from the participants to verify accuracy of calculations, as laboratories only report their final results and are not required to submit detailed calculations as would be common in a traditional collaborative study. Based on this data, the method is not applicable to the quantitation of curcuminoids in tinctures. A future improvement to the method would be preparation of tinctures by mass rather than by volume to improve reproducibility but would require further validation.

This study represents one of the first incremental data collection collaborative studies through a QAP that has been performed. The data indicate that the incremental approach is suitable for evaluating multi-laboratory performance, but several lessons were learned that could improve future studies. First, requesting method information from laboratories prior to sample distribution will help ensure sufficient data is collected for reproducibility calculations. Inclusion of homogenous SRMs or CRMs with known uncertainties in the sample set can help verify laboratory performance. Laboratories should be provided with requirements for calibration standards, either by providing calibration materials to participants or requiring laboratories to use a specified source and lot for the study. When commercial products are being used, sample homogenization of the entire lot prior to distribution is highly recommended. Also, feedback from one laboratory suggested that providing the OMA method information and requirements during enrollment would allow potential participants to acquire materials prior to the sample distribution and may increase laboratory participation. Open communication between participants and study directors is imperative and an additional avenue for data verification would ensure calculation and dilution errors are addressed in a timely fashion.

HorRat values are summarized in Table 7. Values ranging from 0.5 to 2.0 are an indication of method performance that fits within “historical performance” based on traditional collaborative studies (12). As noted above, there are several aspects that were not controlled in this study, which impacted reproducibility including sample heterogeneity, calibration standards, and time. The majority of the very high HorRat values correspond with the materials known to have heterogeneity or preparation issues and the lower performance of BDMC. While HorRat values are suitable in a traditional approach, they may not be applicable to this non-traditional approach with several additional, uncontrolled variables. The method performance should be compared with the requirements for RSD_R_ based on SMPR 2016.003 for curcuminoid analysis while stating the limitations for sample types.

The incremental data approach undertaken with this study was successful in determining method performance for the quantitation of curcuminoids. The data summarized indicate that the method is suitable for use with dried turmeric root powder, turmeric root extract, turmeric capsules, and softgels. Higher variance was observed for some products which is suspected to be due to homogeneity issues of the samples. Based on these findings, at this time the method is not suitable for products with microbeads or tinctures. The results indicate that this method is suitable for the quantitation of individual curcuminoids and should be adopted as a Final Action Official Method for the types of products defined.

## Supplementary Material

Supporting Data and Information

## Figures and Tables

**Table 1.** Descriptions of sample contents for materials used in multi-laboratory validation

Product ID	Description	Additional Ingredients

Sample A	NIST SRM 3299 ground turmeric (*Curcuma longa* L.) rhizome	
Sample B	NIST SRM 3300 curcumin extract of turmeric (*Curucma longa* L.) rhizome	
Sample C	Turmeric root powder	
Sample D	Smoothie mix, dried powder mix containing turmeric root powder, and turmeric root extract	Gota kola leaf (*Centella asiatica*), lemon balm leaf (*Melissa officinalis*), black pepper extract (*Piper nigrum*), cardamom seed powder, allspice berry powder, cinnamon bark powder, ginger root powder, vanilla bean powder, star anise powder, holy basil leaf extract
Sample E	Turmeric root capsule	Vegetable cellulose, vegetable magnesium stearate
Sample F	Turmeric root capsule with black pepper	Turmeric curcumin, black pepper extract, vegetable magnesium stearate, silica, rice powder
Sample G	Multi-component capsule containing turmeric	Alpha-lipoic acid, turmeric root extract, trans-resveratrol, lutein, zeaxanthin
Sample H	Turmeric tincture	
Sample I	Turmeric softgel	Turmeric root extract, black pepper extract, coconut oil, beeswax
Sample J	Turmeric softgel	NovaSOL curcumin (micelle solubilized extract), turmeric root extract, glycerine

**Table 2.** Concentrations of mixed calibrations standards according to OMA 2016.16

	Concentration, *μ*g/mL						
Curcuminoid	Std 1	Std 2	Std 3	Std 4	Std 5	Std 6	Std 7

Curcumin (CUR)	300	225	150	100	50	10	5
Demethoxycurcmin (DMC)	100	80	60	25	10	2.5	1
Bisdemethoxycurcumin (BDMC)	120	75	40	25	10	2.5	1

**Table 3.** Multi-laboratory raw data of curcumin (CUR) content in the test samples

Sample ID	CUR	Lab 1	Lab 2	Lab 3	Lab 4	Lab 5	Lab 6	Lab 7	Lab 8	Lab 9

Sample A	Mean, mg/g	11.3	9.7	12.6	11.4	10.0	9.0	10.8	11.6	10.8
	SD	0.08	0.01	0.4	0.2	0.2	0.2	0.1	0.6	0.2
Sample B	Mean, mg/g	761	836	805	813	784	763	779	863	850
	SD	37	40	56	17	3	15	18	10	9
Sample C	Mean, mg/g	15.6^a^	14.6	15.5	15.4^a^	14.2^a^	13.6	14.9	15.4	15.1^a^
	SD	0.1^a^	0.7	0.8	0.1^a^	0.2^a^	0.1	0.1	0.6	0.1^a^
Sample D	Mean, mg/g	11.2	9.1^a^	8.6^a^	9.9	10.6	8.5^a^	7.5^a^	9.6^a^	9.5
	SD	0.3	0.3^a^	0.6^a^	0.1	2.2	0.3^a^	0.1^a^	0.4^a^	0.3
Sample E	Mean, mg/g	19.6	18.7^a^	18.1^a^	19.3	17.6	16.7^a^	15.5^a^	18.8^a^	18.6
	SD	0.1	0.3^a^	0.5^a^	0.1	0.3	0.2^a^	0.2^a^	0.1^a^	1.1
Sample F	Mean, mg/g	49.5	52.4^a^	50.9^a^	51.6	49.0	51.6^a^	42.0^a,b^	49.3^a^	49.5
	SD	1.0	0.2^a^	1.6^a^	0.6	1.1	2.3^a^	0.3^a^	1.1^a^	1.9
Sample G	Mean, mg/g	–^c^	221^b^	280	305^a^	280^a^	284	294	352^b^	278^a^
	SD	–^c^	62	27	1^a^	30^a^	4	2	8	5^a^
Sample H	Mean, mg/g	725^a,b^	500	466	370^a^	391^a^	430	492	492	253^a^
	SD	70^a^	3	56	3^a^	1^a^	9	4	28	5^a^
Sample I	Mean, mg/g	–^c^	23.6	21.8	24.1^a^	22.7^a^	21.7	23.6	24.5	21.0^a^
	SD	–^c^	1.0	1.3	0.2^a^	1.1^a^	0.3	1.1	1.4	1.4^a^
Sample J	Mean, mg/g	46.8	46.3^a^	43.3^a^	47.3	42.5	41.3^a^	38.0^a^	46.9^a^	48.5
	SD	0.6	0.9^a^	1.5^a^	0.4	0.6	1.0^a^	0.5^a^	1.5^a^	0.5

a Sample was prepared during the second round of analysis.

b Data determined to be outliers based on Cochran’s and Grubb’s tests and removed from reproducibility calculations.

c Data not provided due to laboratory error.

**Table 4.** Multi-laboratory raw data of bisdemethoxycurcurmin (BDMC) content in the test samples

Sample ID	DMC	Lab 1	Lab 2	Lab 3	Lab 4	Lab 5	Lab 6	Lab 7	Lab 8	Lab 9

Sample A	Mean, mg/g	3.16	2.47	3.23	2.96	3.01	2.37	3.05	3.43	2.68
	SD	0.02	0.01	0.6	0.04	0.2	0.03	0.03	0.2	0.01
Sample B	Mean, mg/g	16.3	16.0	18.6	15.3	17.4	15.4	19.1	12.7	20.0
	SD	0.2	0.8	0.9	0.1	0.2	0.4	0.8	0.8	0.1
Sample C	Mean, mg/g	10.0^a^	9.9	11.3	10.4^a^	10.1^a^	9.2	9.9	10.1	9.4^a^
	SD	0.1^a^	0.4	0.5	0.1^a^	0.2^a^	0.1	0.1	0.4	0.1^a^
Sample D	Mean, mg/g	1.39	0.66^a^	1.31^a^	0.64	0.86	0.74^a^	0.58^a^	0.74^a^	0.98
	SD	0.02	0.05^a^	0.14^a^	0.01	0.13	0.01^a^	0.03^a^	0.05^a^	0.03
Sample E	Mean, mg/g	4.79	5.36^a^	4.82^a^	4.33	4.57	3.99^a^	3.95^a^	4.15^a^	4.43
	SD	0.06	0.13^a^	0.15^a^	0.03	0.17	0.04^a^	0.04^a^	0.06^a^	0.15
Sample F	Mean, mg/g	3.48	3.36^a^	3.08^a^	2.97	3.61	2.93^a^	2.56^a^	2.77^a^	3.22
	SD	0.04	0.09^a^	0.15^a^	0.03	0.12	0.05^a^	0.01^a^	0.1^a^	0.08
Sample G	Mean, mg/g	–^c^	8.1	12.6	10.9^a^	10.7^a^	10.1	10.6	13.6	9.0^a^
	SD	–^c^	1.9	0.2	0.1^a^	1.0^a^	0.2	0.1	1.5	0.2^a^
Sample H	Mean, mg/g	26.5^a,b^	236	242	199^a^	210^a^	209	228	225	133^a^
	SD	3.6^a^	1	29	1^a^	1^a^	3	2	27	2^a^
Sample I	Mean, mg/g	–^c^	1.02	1.47^b^	1.07^a^	1.02^a^	1.07	1.18	0.60^b^	1.13^a^
	SD	–^c^	0.04	0.16	0.01^a^	0.05^a^	0.03	0.05	0.04	0.06^a^
Sample J	Mean, mg/g	2.16	1.96^a^	2.28^a^	1.75	1.79	2.21^a^	1.64^a^	1.67^a^	2.76^b^
	SD	0.02	0.02^a^	0.06^a^	0.02	0.01	0.05^a^	0.03^a^	0.07^a^	0.18

a Sample was prepared during the second round of analysis.

b Data determined to be outliers based on Cochran’s and Grubbs tests and removed from reproducibility calculations.

c Data not provided due to laboratory error.

**Table 5.** Multi-laboratory raw data of demethoxycurcurmin (DMC) content in the test samples

Sample ID	BDMC	Lab 1	Lab 2	Lab 3	Lab 4	Lab 5	Lab 6	Lab7	Lab 8	Lab 9

Sample A	Mean, mg/g	3.38	2.95	4.02	3.32	3.03	3.00	3.15	3.37	2.83
	SD	0.03	0.01	0.7	0.04	0.1	0.03	0.04	0.2	0.1
Sample B	Mean, mg/g	116.1	115.7	137.4	122.3	111.6	109.5	105.4	134.4	118.7
	SD	5.4	5.1	14	2.6	0.3	2.1	2.0	5.5	1.3
Sample C	Mean, mg/g	7.68^a^	7.19	8.05	7.78^a^	7.11^a^	6.92	6.87	7.91	7.23^a^
	SD	0.06^a^	0.3	0.1	0.05^a^	0.2^a^	0.06	0.05	0.3	0.08^a^
Sample D	Mean, mg/g	2.33	1.71^a^	2.08^a^	1.84	2.19	2.72^a^	1.56^a^	1.82^a^	1.62
	SD	0.04	0.06^a^	0.2^a^	0.03	0.5	0.06^a^	0.02^a^	0.05^a^	0.05
Sample E	Mean, mg/g	6.35	6.03^a^	6.08^a^	5.76	5.67	5.59^a^	5.27^a^	5.75^a^	5.72
	SD	0.1	0.2^a^	0.06^a^	0.03	0.09	0.05^a^	0.05^a^	0.04^a^	0.2
Sample F	Mean, mg/g	4.71	4.10^a^	4.10^a^	4.13	4.51	4.17^a^	3.54^a^	3.99^a^	3.97
	SD	0.04	0.1^a^	0.3^a^	0.04	0.09	0.08^a^	0.01^a^	0.01^a^	0.09
Sample G	Mean, mg/g	–^b^	44	65	68^a^	59^a^	58	57	81	55^a^
	SD	–^b^	12	9	0.2^a^	6^a^	0.9	0.4	5	1^a^
Sample H	Mean, mg/g	150^a^	237	253	195^a^	199^a^	214	219	233	129^a^
	SD	14^a^	1	30	1^a^	1^a^	4	2	18	2^a^
Sample I	Mean, mg/g	–^b^	4.53	5.28	5.03^a^	4.52^a^	4.45	4.39	4.42	4.34^a^
	SD	–^b^	0.2	0.6	0.05^a^	0.2^a^	0.1	0.2	0.3	0.3^a^
Sample J	Mean, mg/g	11.1	10.3^a^	9.2^a^	10.2	9.7	10.1^a^	9.4^a^	10.1^a^	10.3
	SD	0.2	0.3^a^	0.1^a^	0.1	0.1	0.2^a^	0.2^a^	0.3^a^	0.3

a Sample was prepared during the second round of analysis.

b ‘Data not provided due to laboratory error.

**Table 6.** Comparison of NIST SRM values to multi-laboratory validation data obtained through the NIST QAP incremental data approach

Sample	Curcuminoid	NIST value, mg/g	NIST SD	Multi-lab value, mg/g	Multi-lab SD

NIST SRM 3299 ground turmeric (*Curcuma longa* L.) rhizome	CUR	11.04	0.21	10.8	1.1
	BDMC	2.84	0.05	2.92	0.4
	DMC	3.14	0.06	3.16	0.3
NIST SRM 3300 curcumin extract of turmeric (*Curucma longa* L.) rhizome	CUR	822	11	806	44
	BDMC	18.3	0.5	16.8	2.3
	DMC	117.1	1.1	119	12

**Table 7.** Statistical summary of the curcuminoid multi-laboratory validation evaluated through an incremental data collection approach

Sample ID	Description	Curcuminoid	Content, mg/g	RSDr, %	RSDR, %	HorRat

Sample A	NIST SRM 3299 ground turmeric (*Curcuma longa* L.) rhizome	CUR	10.8	2.4	10.3	2.6
		BDMC	3.0	3.4	9.7	2.8
		DMC	3.2	4.3	7.8	1.6
Sample B	NIST SRM 3300 curcumin extract of turmeric (*Curucma longa* L.) rhizome	CUR	806	3.5	5.5	2.6
		BDMC	16.8	3.5	13.8	3.7
		DMC	119	4.8	9.9	3.6
Sample C	Turmeric root powder	CUR	14.9	2.8	5.1	1.3
		BDMC	10.0	2.8	6.4	1.6
		DMC	7.4	2.3	6.3	1.5
Sample D	Multicomponent smoothie mix containing turmeric, gota kola leaf, lemon balm, black pepper, cardamom, allspice, cinnamon, ginger, vanilla bean, star anise, holy basil	CUR	9.5	9.1	13.1	3.2
		BDMC	0.9	7.9	33.9	5.9
		DMC	1.9	9.2	15.6	3.0
Sample E	Turmeric root capsule	CUR	18.2	2.3	7.7	2.1
		BDMC	4.5	2.4	10.3	2.3
		DMC	5.8	2.1	5.7	1.3
Sample F	Turmeric root capsule with black pepper	CUR	50.4	2.7	3.4	1.1
		BDMC	3.11	2.8	11.3	2.4
		DMC	4.13	3.1	8.4	1.8
Sample G	Multicomponent capsule containing alpha-lipoic acid, turmeric, resveratrol, lutein, zeaxanthin	CUR	287	5.9	6.0	2.5
		BDMC	10.7	8.6	18.1	4.6
		DMC	60.8	9.6	19.2	6.3
Sample H	Turmeric tincture	CUR	424	5.3	20.3	3.2
		BDMC	210	6.6	17.2	2.4
		DMC	203	6.2	20.7	2.9
Sample I	Turmeric softgel with black pepper and coconut oil	CUR	22.9	4.6	6.7	1.9
		BDMC	1.13	6.4	14.8	2.7
		DMC	4.64	6.2	9.0	2.0
Sample J	Turmeric softgel with micelle solubilized curcumin, turmeric root extract, and glycerine	CUR	44.4	2.3	8.1	2.5
		BDMC	1.98	1.97	13.1	2.6
		DMC	10.1	2.0	5.6	1.4
