# Supplementary material for: Determination of Curcuminoids in Turmeric Dietary Supplements by HPLC-DAD: Multi-laboratory Study Through the NIH-ODS/NIST Quality Assurance Program
Source: J AOAC Int. 2020 May 19;103(6):1625–32. doi: 10.1093/jaoacint/qsaa069 (PMC8174018; doi:10.1093/jaoacint/qsaa069)
Supplement: qsaa069_Supplementary_Data [file qsaa069_supplementary_data.docx]

**Determination of curcuminoids in turmeric dietary supplements by HPLC-DAD: Multi-laboratory Study through the NIST Quality Assurance Program**

Mudge, Elizabeth M.; Brown, Paula N.; Rimmer, Catherine A.; Phillips, Melissa M.

**Supporting Information**


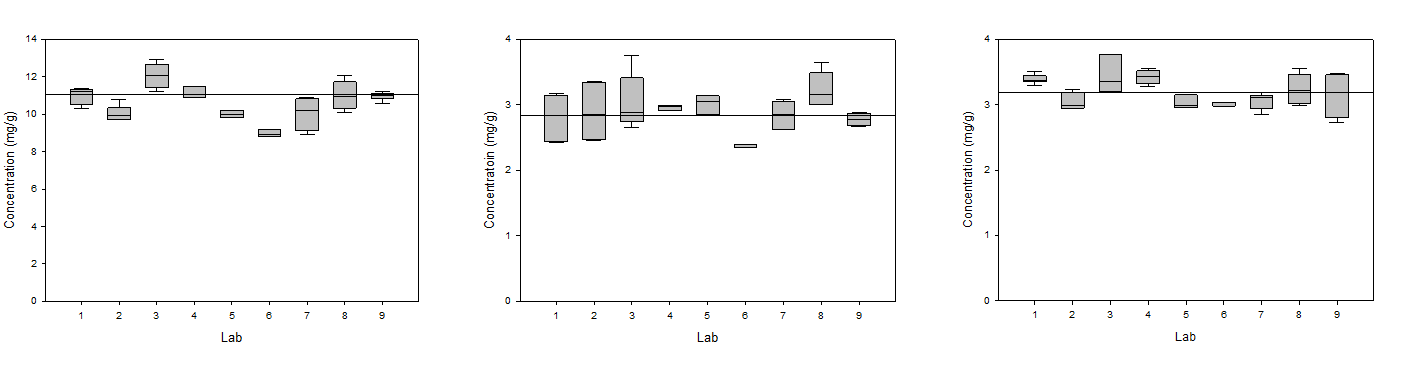


(a)

(b)

(c)

Figure S1. Distribution of (a) curcumin, (b) bisdemethoxycurcumin and (c) demethoxycurcumin in Sample A – NIST SRM 3299. The solid line signifies NIST certified value.


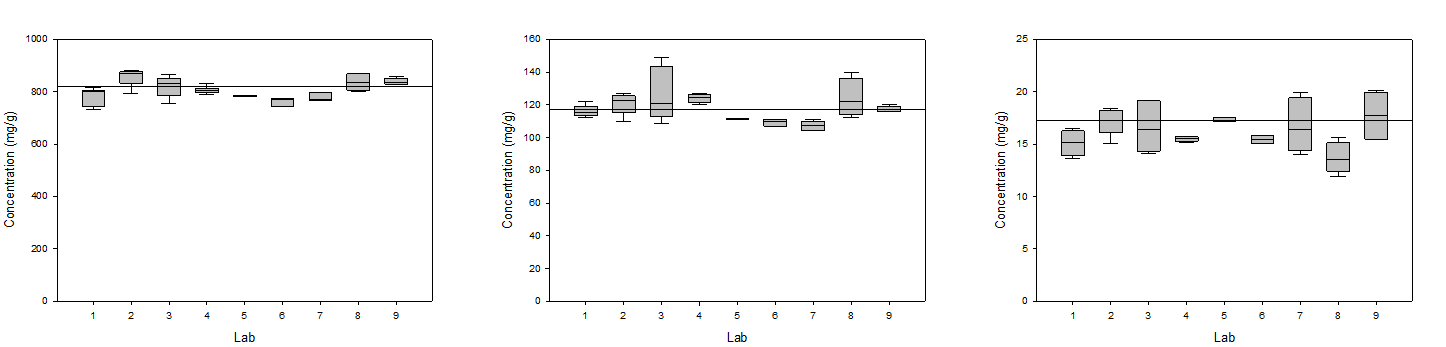


(a)

(b)

(c)

Figure S2. Distribution of (a) curcumin, (b) demethoxycurcumin and (c) bisdemethoxycurcumin in Sample B – NIST SRM 3300. The solid line signifies NIST certified value.


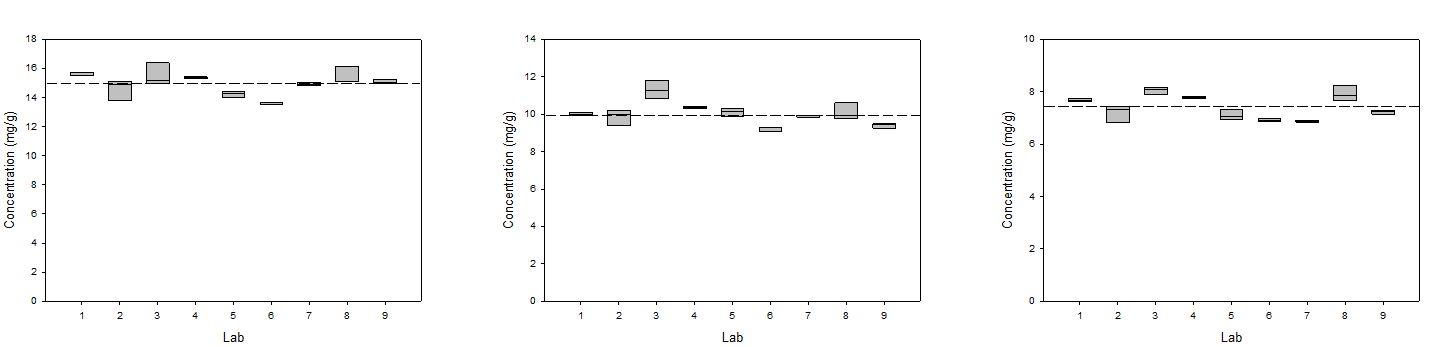


(a)

(b)

(c)

Figure S3. Distribution of (a) curcumin, (b) bisdemethoxycurcumin and (c) demethoxycurcumin measurements obtained for Sample C, turmeric root powder. The dotted line signifies the mean value across the laboratories with outliers removed.


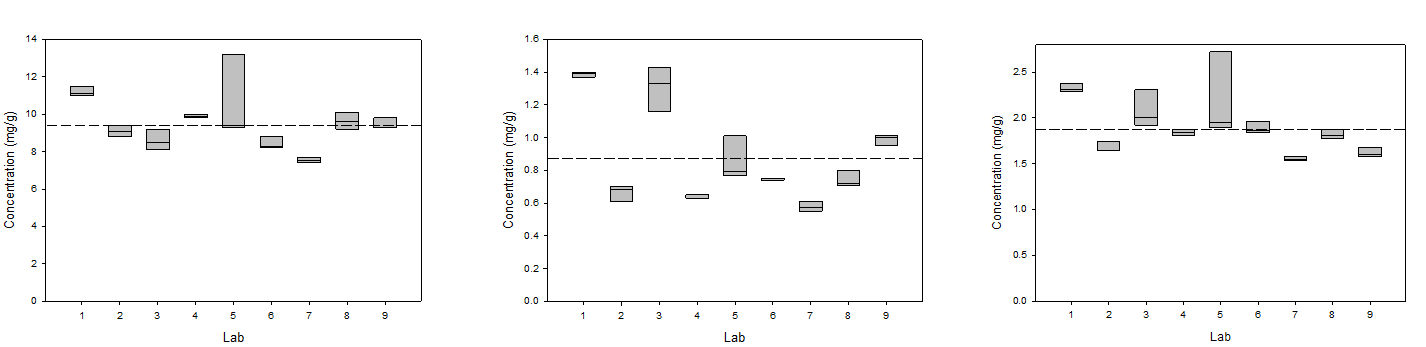


(a)

(b)

(c)

Figure S4. Distribution of (a) curcumin, (b) bisdemethoxycurcumin and (c) demethoxycurcumin measurements obtained for Sample D, Smooothie Additive. The dotted line signifies the mean value across the laboratories with outliers removed.


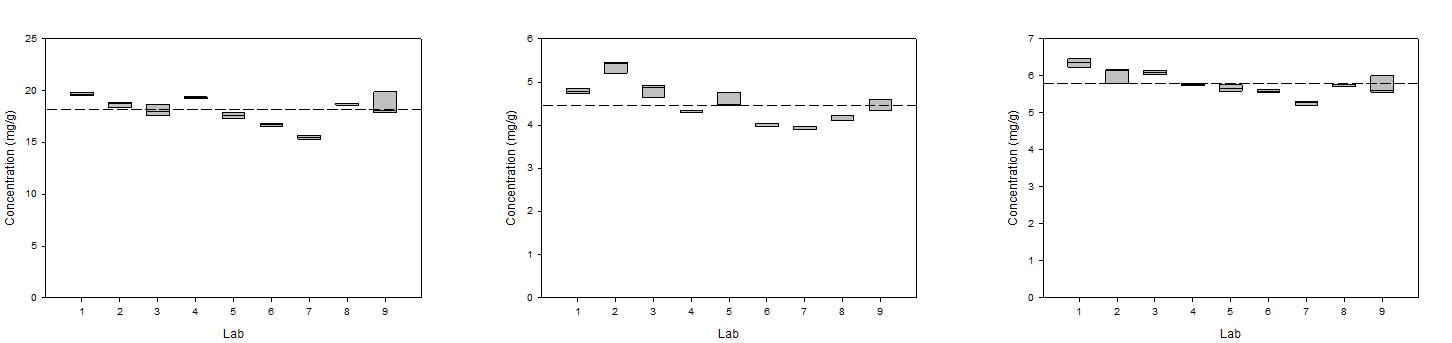


(a)

(b)

(c)

Figure S5. Distribution of (a) curcumin, (b) bisdemethoxycurcumin and (c) bisdemethoxycurcumin measurements obtained for Sample E, Turmeric Root Capsule. The dotted line signifies the mean value across the laboratories with outliers removed.


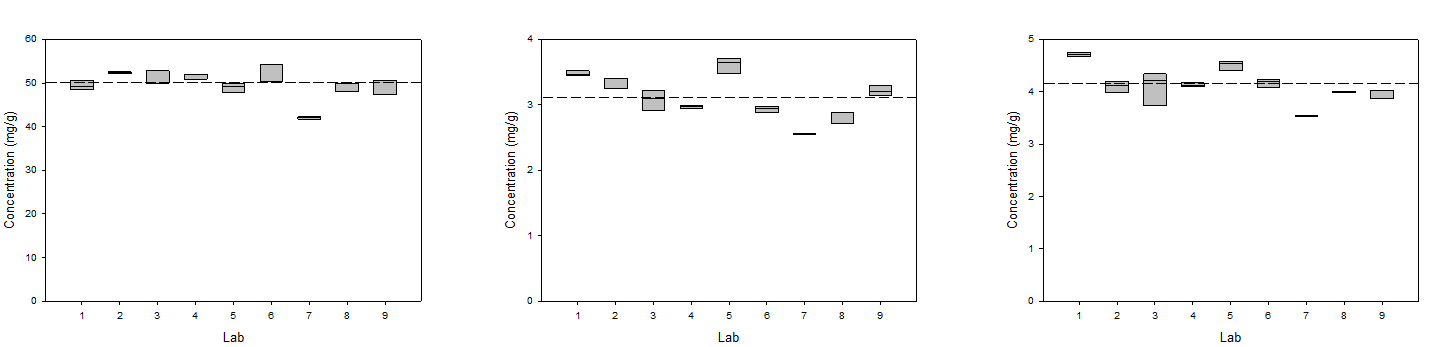


(a)

(b)

(c)

Figure S6. Distribution of (a) curcumin, (b) bisdemethoxycurcumin and (c) demethoxycurcumin measurements obtained for Sample F, Turmeric Root Capsule with black pepper. The dotted line signifies the mean value across the laboratories with outliers removed.


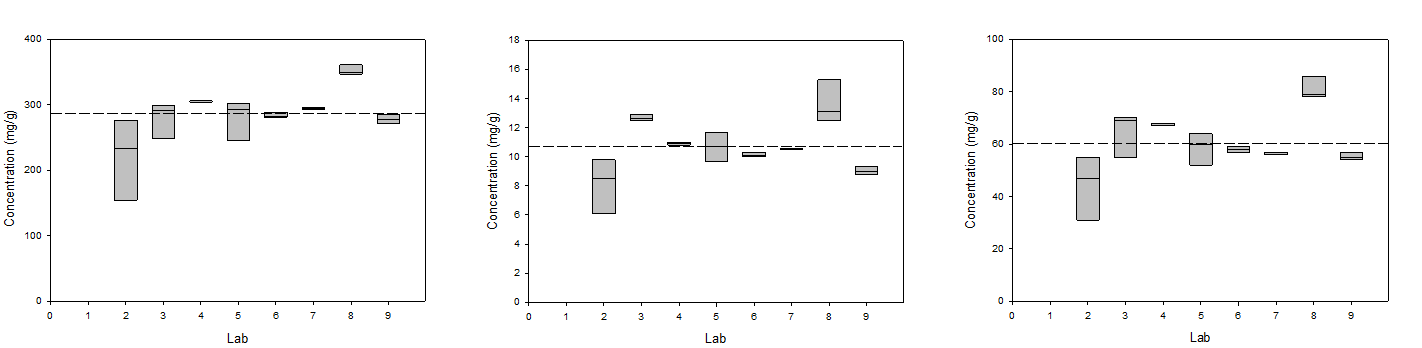


(a)

(b)

(c)

Figure S7. Distribution of (a) curcumin, (b) bisdemethoxycurcumin and (c) demethoxycurcumin measurements obtained for Sample G, Multicomponent turmeric capsule. The dotted line signifies the mean value across the laboratories with outliers removed.


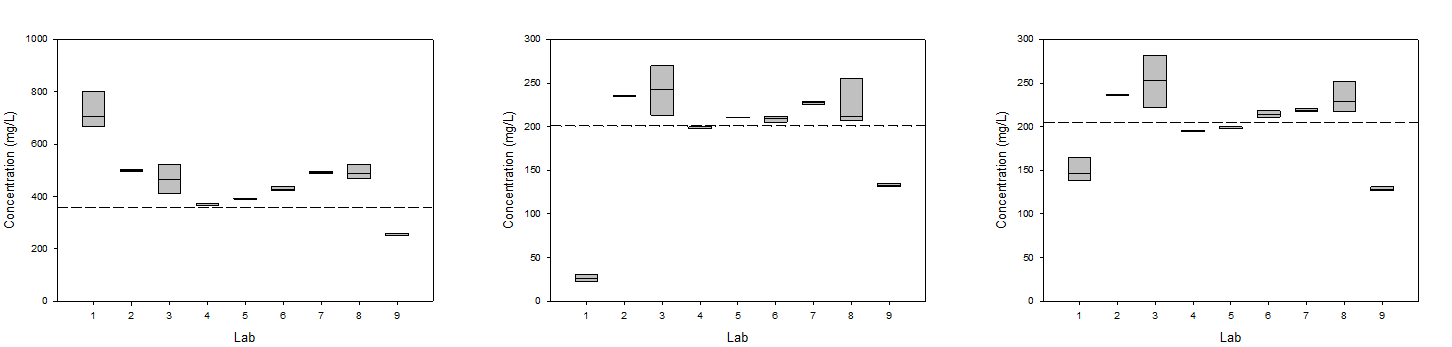


(a)

(b)

(c)

Figure S8. Distribution of (a) curcumin, (b) bisdemethoxycurcumin and (c) demethoxycurcumin measurements obtained for Sample H, turmeric tincture. The dotted line signifies the mean value across the laboratories with outliers removed.


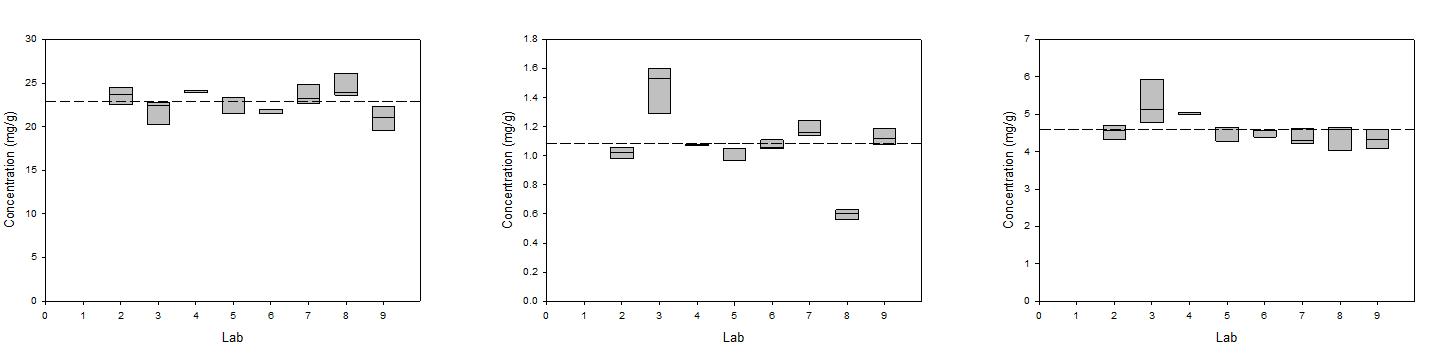


(a)

(b)

(c)

Figure S9. Distribution of (a) curcumin, (b) bisdemethoxycurcumin and (c) demethoxycurcumin measurements obtained for Sample I, Turmeric softgel. The dotted line signifies the mean value across the laboratories with outliers removed.


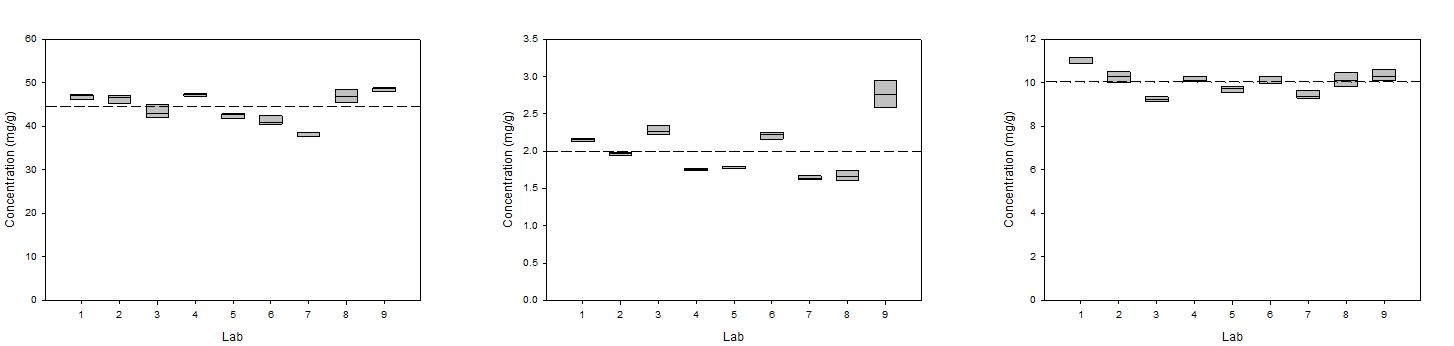


(a)

(b)

(c)

Figure S10. Distribution of (a) curcumin, (b) bisdemethoxycurcumin and (c) demethoxycurcumin measurements obtained for Sample J, Turmeric softgel with black pepper and coconut oil. The dotted line signifies the mean value across the laboratories with outliers removed.
